# Diagnostic Pearls and Clinical Implications of Prekallikrein Deficiency

**DOI:** 10.7759/cureus.8349

**Published:** 2020-05-29

**Authors:** Hassaan Yasin, Muhammad Omer Jamil, Lance A Williams, III

**Affiliations:** 1 Hematology/Oncology, Joan C. Edwards School of Medicine, Marshall University, Huntington, USA; 2 Transfusion Medicine/Pathology, University of Alabama at Birmingham, Birmingham, USA

**Keywords:** fletcher factor deficiency, prekallikrein deficiency, hemostasis, blood coagulation, partial thromboplastin time.

## Abstract

Prekallikrein (PK) deficiency is extremely rare, and manifestations are not well characterized due to a small number of cases reported and the lack of scientific clarity about its role in clot formation in vivo. Here, we report a case of a 64-year-old male, with no known history of abnormal bleeding, who scheduled to undergo deep brain stimulator placement for control of his Parkinson’s disease. During pre-procedure testing, activated partial thromboplastin time (PTT) was found to be prolonged at 146 seconds. Mixing studies were suggestive of a coagulation factor deficiency. His PTT characteristically became shorter with prolonged incubation, providing a clue at testing for PK levels, which were found to be severely low. He, subsequently, underwent surgery without any complications. Our case further highlights the clinical pearls for diagnosis and further endorses that these patients can safely undergo surgical procedures without the need for plasma transfusions or factor concentrate usage.

## Introduction

Prekallikrein (PK) is a glycoprotein involved in the intrinsic pathway of the coagulation system. PK deficiency is extremely rare, with only a small number of cases reported. Thus, manifestations of PK deficiency are not well characterized. Here, we report a case of a patient with PK deficiency scheduled to undergo a neurosurgical procedure.

## Case presentation

A 64-year-old African American male was admitted to the hospital with a syncopal episode. His past medical history was significant for Parkinson’s disease, mild cognitive impairment, hypertension, and diabetes mellitus type 2. Past surgical history was notable for bilateral rotator cuff repair and appendectomy. There was no reported personal or family history of easy bruising, nose bleeds, bleeding gums, abnormal bleeding after surgery, thrombosis, or abnormal coagulation tests. His home medications did not include any antiplatelet or anticoagulation medications. He reported occasional alcohol use, but denied smoking or illicit drug use.

His admission vital signs were normal, and his physical exam was unremarkable with the exception of generalized rigidity and impaired balance. The only remarkable finding on the initial labs was an elevated partial thromboplastin time (PTT) of 40 seconds (25-35 seconds).

He was diagnosed with syncope secondary to dysautonomia and he was scheduled for deep brain stimulator placement. Since the PTT was mildly elevated, coagulation labs were repeated. He was also started on subcutaneous Lovenox for deep venous thrombosis prophylaxis. 

Repeat coagulation labs revealed a normal prothrombin time/international normalized ratio (PT/INR) of 12.9 seconds/0.9 (11-14 seconds/<1.1) but a prolonged PTT of 55 seconds. The test was performed the same day, and the results showed a PT/INR of >100 seconds/>12 and a PTT >200 seconds. Coagulation times when repeated on the same sample revealed a PT/INR of 12.1 seconds/0.9 and an elevated PTT of 146 seconds. This prompted a new sample to be sent out again. The coagulation test results at this time showed a PTT of 46 seconds. Given the unexplained variation in results on the samples, heparin contamination and sample mix ups were ruled out. The lab technicians also recalibrated the machines and control, but continued to get different results for PTT even when sent to different coagulation labs within our health system.

The hematology/oncology service was consulted, and mixing studies were performed. The elevated PTTs corrected from 146 to 30 seconds and from 69 to 30 seconds after a 1:1 mix with normal plasma, indicating a factor deficiency as the likely cause of the prolonged PTT. Von Willebrand factor antigen, ristocetin cofactor activity, factor VIII, factor IX, factor XI, and factor XII levels were all found to be within normal limits. In the absence of a deficiency in the intrinsic pathway coagulation factors, and because of the unexplained sample to sample variation in the PTT, PK deficiency was suspected. Therefore, a PK level was obtained from a reference lab which revealed an activity of less than 1%, solidifying the diagnosis of PK deficiency. The patient was cleared for surgery by hematology/oncology and he underwent the procedure without any bleeding complications.

## Discussion

PK is a glycoprotein synthesized in the liver with its gene located on chromosome 4 [1]. It circulates in the plasma bound to high molecular weight kallikrein (HMWK) with only 20% in free form. The inactive PK is converted to the active kallikrein by factor XIIa and also by the proteins derived from the endothelial cells [1]. Kallikrein, in turn, regulates activation of factor XII to XIIa and the formation of bradykinin from HMWK before it is rapidly degraded in plasma by alpha-2 microglobulin and C1 esterase. Although not well characterized, PK is also believed to be implicated in the activation of plasminogen, decreasing blood pressure and increasing capillary permeability. There is data on both decreased and normal fibrinolytic activity with PK deficiency, but no clinically significant effects have been reported so far on blood pressure or capillary permeability [2]. 

PK deficiency was first reported in the Fletcher family in Kentucky in 1965 by Hathaway et al. [3]. In 1973, Wuepper et al. showed that the “Fletcher factor” was actually PK [4]. It is an autosomal recessive disorder with more than seven different types of mutation identified by molecular genetic analysis [5]. PK deficiency has been classified as type I and type II based on the level of activity and antigen. Type I, seen in 80% of cases, exhibits deficiency in both activity and antigen, whereas type II is associated with deficient activity and normal antigen levels.

The exact prevalence of PK deficiency is unknown. So far less than 100 cases have been reported in the literature and it is likely that it is underreported as it is not associated with bleeding, thrombosis or any other severe clinical symptoms [6]. In the majority of cases that have been reported, patients underwent numerous procedures without any bleeding complications [7]. Although case reports exist where patients with bleeding were found to have PK deficiency, almost all of them are more than 20 years old when coagulation testing may have been incomplete. Some other case reports have also attributed PK deficiency to cases with arterial and venous thrombosis, but recent studies have suggested that most of these patients had other risk factors for thrombosis [8]. Recent clinical experience and studies make it evident that PK deficiency manifests the same way as factor XII deficiency, does not lead to bleeding, and does not prevent thrombosis.

PK deficiency largely presents as a marked elevation in PTT with a normal PT/INR and thrombin time (TT). Bleeding, however, is not seen as factors downstream (factor XII, factor XI, etc.) can be auto-activated or activated by other factors like thrombin or factor II. The PTT, therefore, decreases and can normalize if incubated long enough, thus explaining the sample to sample variability that is often noticed with PK deficiency, as highlighted in our patient [9]. Additionally, variation in PTT prolongation may be seen depending on the type of reagent used, which include silica, ellagic acid, and kaolin [10]. PK’s activity is tested similarly to other factors. Its antigen level can be tested by enzyme-linked immunosorbent assay (ELISA).

The prognosis of a patient with PK deficiency is the same as the normal patient population; therefore, do not require specific therapy. Its clinical significance is in patients on heparin treatment being monitored with the PTT since PK deficiency falsely elevates the PTT. Thus, anti-Xa level monitoring is recommended. Those with thrombosis are treated according to the guidelines for normal patients with similar thrombosis or thrombosis risk.

## Conclusions

This case highlights the diagnostic challenge of PK deficiency and the diagnostic pearls that can help clinicians quickly hone in on the correct diagnosis. Our case further corroborates the belief that despite extreme prolongation of the PTT, patients with PK deficiency do not exhibit signs or symptoms of abnormal bleeding, and are not at increased risk for bleeding compared to the general population when undergoing a surgical intervention. Therefore, neither transfusion of plasma nor infusion of factor concentrates is necessary for these patients. Lastly, the monitoring of heparin therapy, using the anti-Xa instead of the PTT, is an important clinical consequence of PK deficiency.
